# Comparative Pulmonary Toxicity of Two Ceria Nanoparticles with the Same Primary Size

**DOI:** 10.3390/ijms15046072

**Published:** 2014-04-10

**Authors:** Lu Peng, Xiao He, Peng Zhang, Jing Zhang, Yuanyuan Li, Junzhe Zhang, Yuhui Ma, Yayun Ding, Zhenqiang Wu, Zhifang Chai, Zhiyong Zhang

**Affiliations:** 1 School of Biological Science and Engineering, South China University of Technology, Guangzhou 510006, China; E-Mail: penglu10@126.com; 2 CAS Key Laboratory for Biomedical Effects of Nanomaterials and Nanosafety, CAS Key Laboratory of Nuclear Radiation and Nuclear Energy Technology, Institute of High Energy Physics, Chinese Academy of Sciences, Beijing 100049, China; E-Mails: pengzhang@ihep.ac.cn (P.Z.); liyy@ihep.ac.cn (Y.L.); mayh@ihep.ac.cn (Y.M.); dingyy@ihep.ac.cn (Y.D.); chaizf@ihep.ac.cn (Z.C.); 3 Beijing Synchrotron Radiation Facility, Institute of High Energy Physics, Chinese Academy of Sciences, Beijing 100049, China; E-Mail: jzhang@ihep.ac.cn; 4 College of Environmental Science and Engineering, Ocean University of China, Qingdao 266100, China; E-Mail: zhangjz@ihep.ac.cn

**Keywords:** nano-ceria, pulmonary toxicity, agglomerates, size distribution, surface chemistry

## Abstract

Ceria nanoparticles (nano-ceria) have recently gained a wide range of applications, which might pose unwanted risks to both the environment and human health. The greatest potential for the environmental discharge of nano-ceria appears to be in their use as a diesel fuel additive. The present study was designed to explore the pulmonary toxicity of nano-ceria in mice after a single exposure via intratracheal instillation. Two types of nano-ceria with the same distribution of a primary size (3–5 nm), but different redox activity, were used: Ceria-p, synthesized by a precipitation route, and Ceria-h, synthesized by a hydrothermal route. Both Ceria-p and Ceria-h induced oxidative stress, inflammatory responses and cytotoxicity in mice, but their toxicological profiles were quite different. The mean size of Ceria-p agglomerates was much smaller compared to Ceria-h, thereby causing a more potent acute inflammation, due to their higher number concentration of agglomerates and higher deposition rate in the deep lung. Ceria-h had a higher reactivity to catalyzing the generation of reactive oxygen species (ROS), and caused two waves of lung injury: bronchoalveolar lavage (BAL) inflammation and cytotoxicity in the early stage and redox-activity-evoked lipid peroxidation and pro-inflammation in the latter stage. Therefore, the size distribution of ceria-containing agglomerates in the exhaust, as well as their surface chemistry are essential characteristics to assess the potential risks of using nano-ceria as a fuel additive.

## 1. Introduction

Cerium oxide nanoparticles (nano-ceria) are engineered nanomaterials (ENMs) that possess unique catalytic, optical and anti-oxidant properties [1]. The widespread use of nano-ceria in industry has raised occupational and environmental health concerns over the potential risks of nano-ceria exposure. Therefore, nano-ceria are identified by the Organisation for Economic Co-operation and Development as one of the 13 priority listed representative ENMs for immediate testing [2].

Nano-ceria are used increasingly as an additive to improve the burning efficiency of fuels, reducing fuel consumption, greenhouse gases and particle numbers in vehicle exhaust [3]. Nanoscale ceria could be found in the soot matrix and, thereby, be released into the environment [4]. By using a modeling method, Park *et al.* predicted that receptors in the car passenger area of a street canyon would be exposed to ceria at a concentration up to 80 ng/m_3_ in the worst case [5]. The combusted nano-ceria would interact with the carbonaceous aggregates or other co-pollutants in the exhaust, thereby exhibited a toxicological profile different from nano-ceria alone. Therefore, risk-assessment studies should be conducted based on the combusted particulates emitted from engines using nano-ceria-based fuel additives [6]. Furthermore, the toxicity of the combusted nano-ceria should be correlated to their physicochemical properties, so that researchers are able to predict potential risks and design ceria-based additives with minimum toxicity.

However, the physicochemical properties of nano-ceria would be changed during combustion, and the details of the changes remain unknown. For example, the reported crystallite size of nano-ceria in diesel exhaust were quite controversial: ranging from 1–3 nm [6], 5–7 nm [7], up to about 43 nm [4]. There is very limited knowledge about the other physicochemical properties of the combusted ceria (e.g., surface chemistry). The gap makes it difficult to understand the mechanisms underlying the toxic impact from the combusted nano-ceria when considering the hazard posed by the use of nano-ceria as a fuel additive. Alternatively, toxicological assessments of a series of nano-ceria with diverse, but well-defined, nano-properties were conducted in previous studies [5,8,9] to relate the physicochemical properties of nano-ceria to their toxicity.

Recent literature has shown that nano-ceria exposure is associated with pulmonary injury, leading to oxidative stress, inflammation and cytotoxicity [9–11]. In our previous study, mice were exposed to 0.04, 0.4, 4 and 40 μg of nano-ceria with a mean diameter of 6.6 nm (6.6-Ceria) to explore the pulmonary toxicity of nano-ceria after an acute intratracheal instillation [8]. It was indicated that only the exposure to 40 μg of 6.6-Ceria caused changes in the differential bronchoalveolar lavage (BAL) fluid (BALF) cell counts and BALF lactate dehydrogenase (LDH) activity as an acute inflammatory reaction; the cell counts returned to normal at seven days post-instillation (PI) and did not cause oxidative damage to the lungs; the particle overload in pulmonary macrophages led to multifocal acute alveolitis and pulmonary granulomas at seven days PI and recovered at 28 days PI.

The size of ENMs plays a key role in determining the toxicity of ENMs [12]. Compared with their larger counterparts, smaller ENMs are of a larger number concentration at the same mass concentration and have a higher surface energy and reactivity, greater mobility and greater resistance to systemic clearance. In the present work, we tried to test whether exposure to nano-ceria sized 3–5 nm could cause more severe pulmonary toxicity than 6.6-Ceria did. Mice were intratracheally exposed to two kinds of nano-ceria were are of a similar size, but have different redox activity, and the pulmonary toxicities in terms of oxidative damage, inflammation and histopathological changes were assessed.

## 2. Results

### 2.1. Characterization of Nano-Ceria

**Figure 1.**
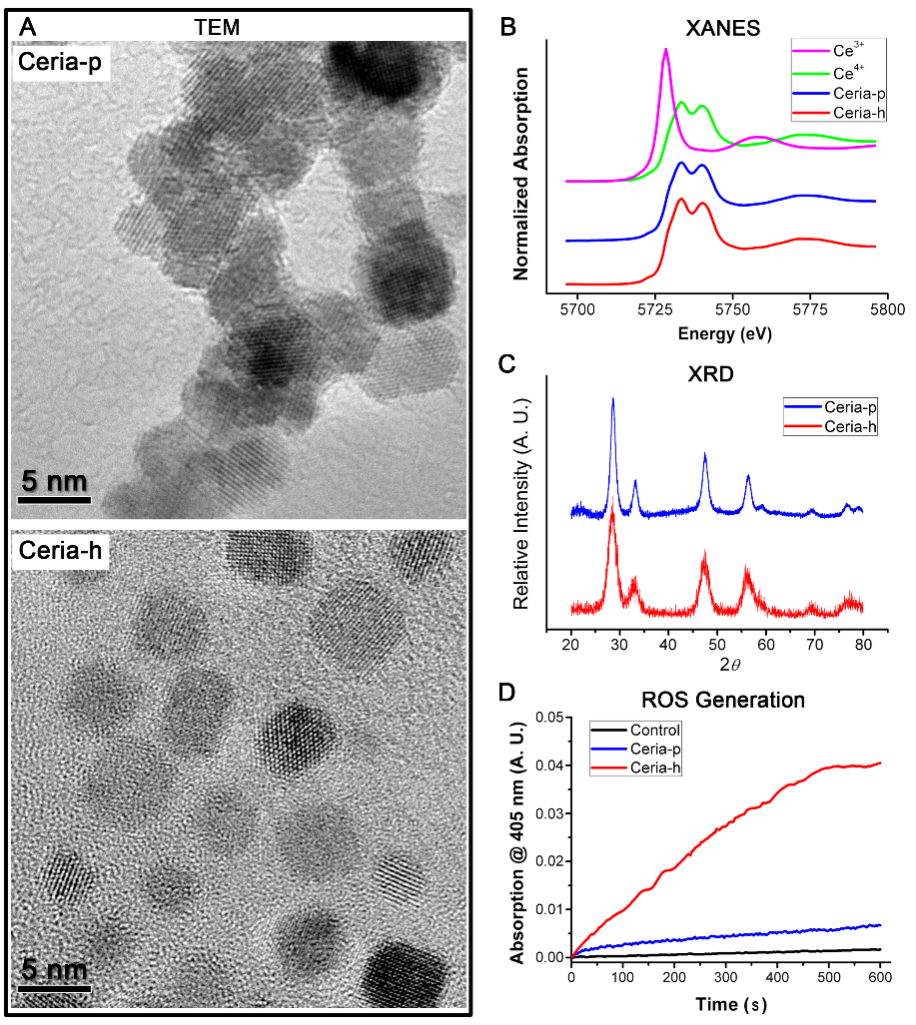
The physicochemical characteristics of Ceria-p (synthesized via precipitation) and Ceria-h (synthesized via the hydrothermal method). (**A**) transmission electron microscopy (TEM) images of Ceria-p and Ceria-h; (**B**) the X-ray absorption near-edge structure (XANES) spectra of Ceria-p and Ceria-h; (**C**) the X-ray diffraction (XRD) patterns of Ceria-p and Ceria-h; and (**D**) the generation of reactive oxygen species (ROS) catalyzed by Ceria-p and Ceria-h in the presence of hydrogen peroxide; and the form of 2,2′-azinobis-(3-ethylbenzthiazoline-6-sulfonic acid) cation radical (ABTS^+#^) was photometrically determined at 405 nm.

The two types of nano-ceria were synthesized via precipitation (Ceria-p) or the hydrothermal (Ceria-h) method, respectively. The morphologies of Ceria-p and Ceria-h were studied by transmission electron microscopy (TEM), and the size distributions of their primary particles were very similar, within a range of 3–5 nm (Figure 1A). However, dynamic light scattering (DLS) results showed that the hydrodynamic size of Ceria-p and Ceria-h in phosphate buffer (PBS) were 313 ± 30 nm *vs.* 1731 ± 165 nm. Therefore, particle agglomeration would occur after Ceria-p and Ceria-h were instilled into the airway, and Ceria-h agglomerates had a significantly larger size distribution compared to Ceria-p agglomerates. X-ray absorption near-edge structure (XANES) and X-ray diffraction (XRD) measurement demonstrated that Ceria-p and Ceria-h were the same in the oxidation state of cerium and in the crystalline peaks (Figure 1B,C). In the present work, Ceria-p and Ceria-h both had no detectable fraction of Ce^3+^ (Figure 1B). However, their redox activities were quite different: the generation of reactive oxygen species (ROS) was catalyzed more efficiently by Ceria-h than by Ceria-p.

### 2.2. Cytological and Biochemical Assessments of BALF

**Figure 2.**
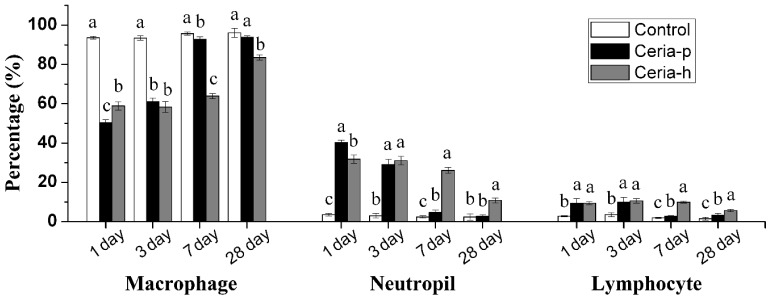
Percentages of macrophage, neutrophil and lymphocyte in bronchoalveolar lavage fluid (BALF) after intratracheal instillation of Ceria-p and Ceria-h. Values are represented as the mean ± standard deviation (SD) of at least 5 mice. At each time point, columns labeled with different lowercase letters are significantly different at *p* < 0.05 using Tukey’s test.

The intratracheal instillation of either Ceria-p or Ceria-h had no statistically significant effect on the body weight of mice at any time point. However, both treatments caused significant lung inflammation (Figure 2). Cell differential counts in BALF revealed increases in the percentage of neutrophils at 1, 3 and 7 days PI, supporting the presence of pulmonary inflammation. At 1 day PI, the neutrophilic inflammatory responses to Ceria-p were more potent when compared to Ceria-h; but the influx of neutrophils in BALF caused by Ceria-p decreased faster, returning to normal at 28 days PI. Ceria-h triggered substantial inflammation in BALF at 1 day PI, followed by a moderate recruitment of neutrophils throughout the whole experimental period.

**Figure 3.**
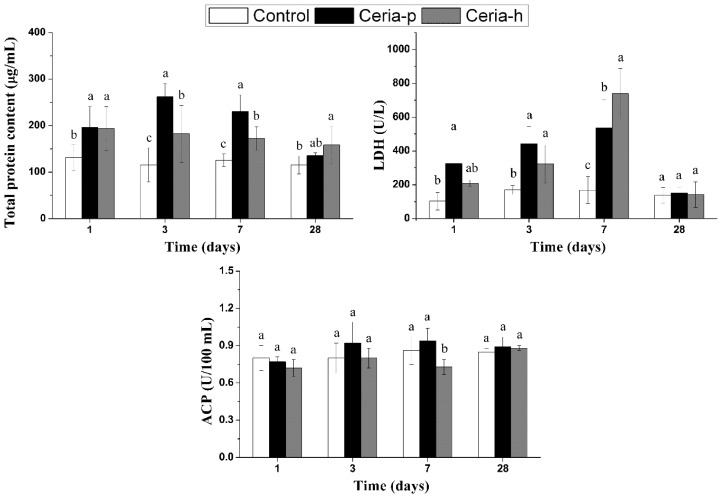
The levels of total protein concentration, lactate dehydrogenase (LDH) and acid phosphatase (ACP) in BALF after intratracheal instillation of Ceria-p and Ceria-h. Values are represented as the mean ± SD. At each time point, columns labeled with different lowercase letters are significantly different at *p* < 0.05 using Tukey’s test.

Both Ceria-p and Ceria-h exerted cytotoxicities against BALF cells, as evidenced by an increased total protein concentration and LDH activity in BALF. The total protein concentration in BALF showed a significant increase immediately after the instillation in both treatment groups (Figure 3). Ceria-p induced higher BALF protein content at 3 and 7 days PI than Ceria-h did; but at 28 days PI, no significant effect was found in the Ceria-p group, while a modest increase in BALF protein could still be seen in the Ceria-h group. LDH leakage caused by Ceria-p instillation could be seen at 1, 3 and 7 days PI and recovered at 28 days PI. Ceria-h caused LDH leakage could only be seen at 3 and 7 days PI. Both treatments had little effect on the level of acid phosphatase (ACP) in BALF, except for a reduction caused by Ceria-h at 7 days PI.

### 2.3. Biochemical Analysis of Lung Homogenate

**Figure 4.**
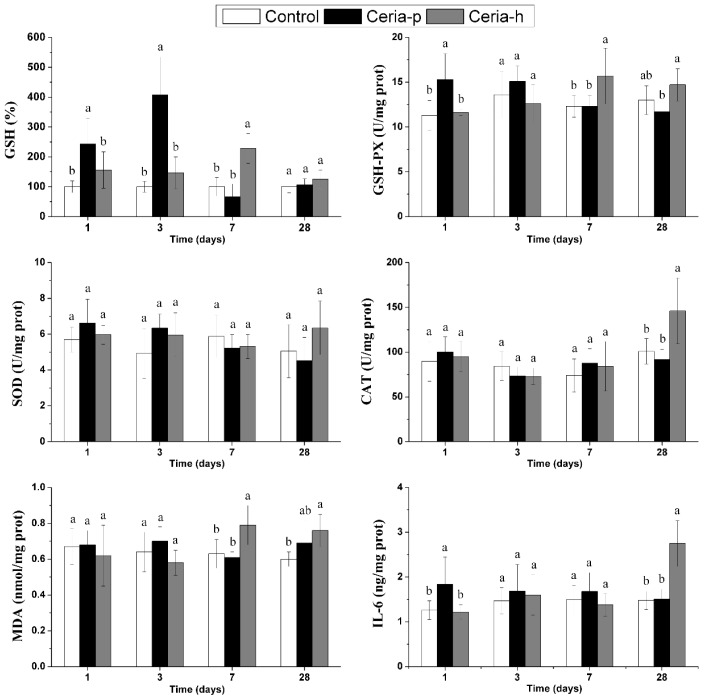
Levels of glutathione (GSH), glutathione peroxidase (GSH-Px), superoxide dismutase (SOD), catalase (CAT), malondialdehyde (MDA) and interleukin-6 (IL-6) in lung homogenates after intratracheal instillation of Ceria-p and Ceria-h. Values are represented as the mean ± SD. At each time point, columns labeled with different lowercase letters are significantly different at *p* < 0.05 using Tukey’s test.

The supernatants of lung homogenates were collected to determine the oxidative stress and pro-inflammatory responses following the acute instillation of either nano-ceria (Figure 4). In the present study, the pulmonary response to the oxidative stress was evaluated by the detection of intracellular markers, including reduced glutathione (GSH), glutathione peroxidase (GSH-Px), superoxide dismutase (SOD) and catalase (CAT). The results suggest that the antioxidant defenses were activated during the acute phase of Ceria-p exposure (1 and 3 days PI), as evidenced by increased levels of GSH and GSH-Px, and these effects could not be measured later. There is no evidence that the anti-oxidative capacities were exhausted during Ceria-p exposure, since no lipid peroxidation was found. The level of pro-inflammatory cytokine interleukin-6 (IL-6) exhibited an immediate/early response to Ceria-p: it was upregulated at 1 day PI and returned to normal later.

In contrast, Ceria-h exposure did not deliver any oxidative stress and pro-inflammatory effects to lung tissue immediately after the instillation. However, delayed increases of CAT, GSH-Px, GSH and IL-6 were found during the later stages of the experiment (7 and/or 28 days PI). In spite of the enhanced anti-oxidative capacity, Ceria-h exposure resulted in oxidative damage at 7 and 28 days PI, implying an unbalance between the oxidative stress and anti-oxidative defenses.

### 2.4. Histopathological Evaluation

**Figure 5.**
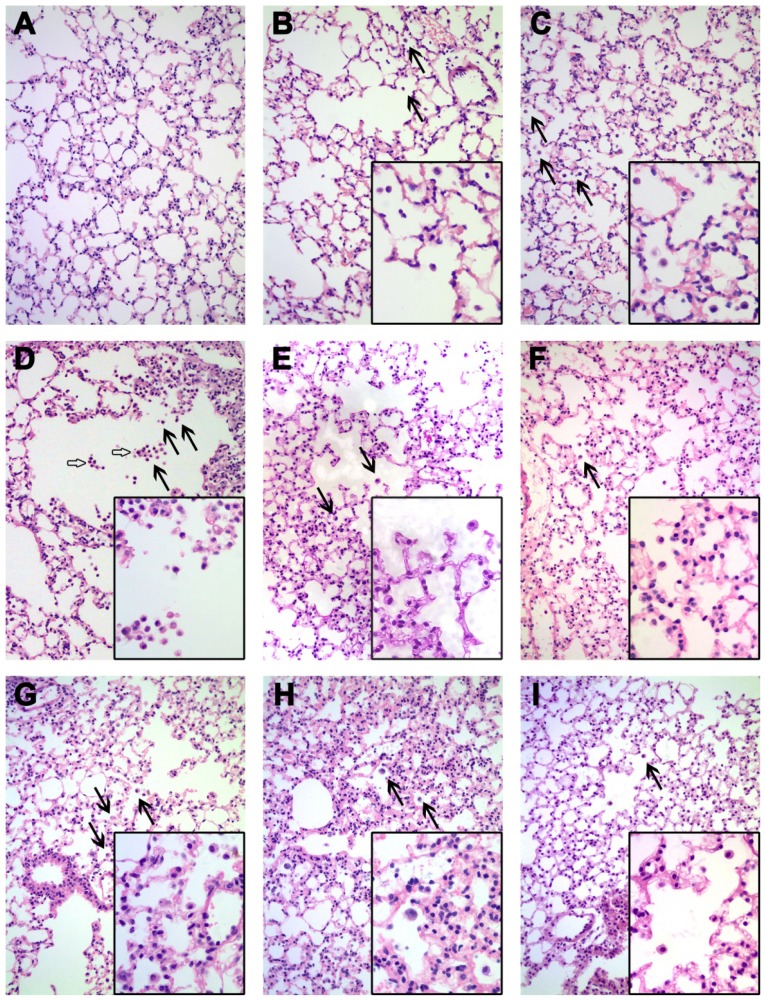
Light micrographs of hematoxylin-eosin stained lung tissue slices after intratracheal instillation of Ceria-p and Ceria-h. (**A**) The lung of mice from the control; (**B**–**E**) the lung of mice exposed to Ceria-p at 1, 3, 7 and 28 days post-instillation (PI), respectively; (**F**–**I**) the lung of mice exposed to CeO_2_-h at 1, 3, 7 and 28 days PI; magnification: original ×200, inset ×400; solid arrow: particle-laden macrophages; hollow arrow: granulomas.

The histopathological changes in lung were evaluated with hematoxylin-eosin (HE) staining in Figure 5. There were pathological changes in the lung tissues of both treatments over the whole experimental period. In the Ceria-p group, particle-laden macrophages could be found at 1 and 3 days PI, increased in number at 7 days PI and then gradually recovered at 28 days (Figure 5B–E); pulmonary granulomas were only occasionally found at 7 days PI, at the peripheral area of the lungs (Figure 5D). While in the Ceria-h group, there were relative less particle-laden macrophages throughout the test, and no further pathological lesion was identified (Figure 5F–I).

## 3. Discussion

Nano-ceria have recently gained a wide range of applications, which might pose unwanted risks to both the environment and human health. The greatest potential for the environmental discharge of nano-ceria appears to be in their use as a diesel fuel additive. According to the prediction by Park *et al.*, the commercial use of nano-ceria will increase the air-borne nano-ceria, to a level of 80 ng/m^3^ in the worst case [5]. In the present work, mice were exposed to 40 μg of nano-ceria via a single intratracheal instillation, corresponding to 10,000 times the maximum daily exposure dose for mice [8].

Although the toxic mechanisms for inhaled ENMs were not very clear, the results of older toxicological and epidemiological studies with airborne fine or ultrafine particles can be viewed as the basis for the expanding field of nanotoxicology [13]. Due to their small size, inhaled ENMs could be transported into the deep lung and could not be removed efficiently, leading to particle-overload in lung. In the present work, particle-laden macrophages could be found in the lungs instilled with either Ceria-p or Ceria-h during the whole experimental period. Our results implied that the pulmonary toxicity of nano-ceria was associated with lung particle-overload.

Studies in rodents imply that the excessive burden of insoluble particles of low cytotoxicity would lead to pulmonary inflammation [14]. Oyabu *et al.* reviewed the reports focusing on particle-overload in lung and concluded that a dose that can induce inflammation is between one and 5 mg/kg [15]. Our previous work also showed that the intratracheal instillation of nano-ceria (with a mean size of 6.6 nm) at a dose of ~1.5 mg/kg (40 μg per mouse) induced moderate and transient inflammation in mouse lung, which recovered by four weeks, whereas 0.15 mg/kg did not cause any inflammation. In the present work, neutrophil inflammation was induced by the exposure to either Ceria-p or Ceria-h at a dose of 1.5 mg/kg (40 μg per mouse). The acute intratracheal instillation of Ceria-p and Ceria-h also influenced the total protein content and LDH leakage in the BALF and induced oxidative stress and inflammatory response in the lung homogenates.

However, there were some differences between the toxicological profiles of Ceria-p and Ceria-h. In general, intratracheal instillation of Ceria-p induced transient inflammatory responses in both BAL cells and lung tissue at the early stage of exposure (one and/or three days PI), which recovered at 7 or 28 days PI. Ceria-h induced initially minor, but more persistent inflammation in BAL cells and delayed lipid peroxidation and pro-inflammation in lung tissue at 28 days PI.

The differences in the initial inflammatory responses may be due to the different agglomeration state of Ceria-p and Ceria-h. The size of agglomerates would determine their deposition in the lung, their ability to cross biological barriers and their cellular internalization [12]. Noël *et al.* found that the pulmonary toxicity of ENMs might depend not solely on the dimension of the ENMs, but also on the dimension of the ENMs agglomerates [16]. However, in their study, large nano-TiO_2_ agglomerates (>100 nm) caused acute inflammatory responses; small ones (<100 nm) exerted both cytotoxicity and oxidative stress without apparent neutrophil influx into the airways, because agglomerates smaller than 100 nm could easily escape the pulmonary clearance via phagocytosis [12,17]. In the present work, Ceria-p agglomerates and Ceria-h agglomerates were both larger than 100 nm (313 ± 30 and 1731 ± 165 nm, respectively). Therefore, macrophage phagocytosis was activated in both treatments. Due to the smaller size of Ceria-p agglomerates, there was a larger number concentration of agglomerates in the Ceria-p exposure. Moreover, smaller agglomerates had a higher deposition rate in the deep lung; particle-laden macrophages and granulomas could even be found at the peripheral area of the lungs instilled with Ceria-p. Therefore, Ceria-p caused a more potent particle-overload inflammation at the early stage of exposure.

In the lungs instilled with Ceria-h, oxidative damage was found at seven and 28 days PI, and a pro-inflammatory effect was found at 28 days PI. These delayed responses seemed not to be attributed exclusively to the excessive ENMs burdens in the lung, as particle-laden macrophages were less observed in the Ceria-h treated mice than in the Ceria-p group. ROS generation is identified as one of the major mechanisms by which inhaled ENMs exert adverse biological effects [18,19]. We speculated that the retention of Ceria-h in lung and their capability to generate ROS might also be involved in the pulmonary toxicity of Ceria-h. Although Ceria-h tended to agglomerate, the agglomeration would be gradually reduced in the airway due to the interactions with surfactant proteins or vascular proteins (in the case of a breakdown of the integrity of the alveolar-capillary barrier caused by ENMs exposure) [1,20]. Deagglomerated Ceria-h might be deposited in the BAL area or translocated into the lung interstitium, where it catalyzes the generation of ROS and increases the risk of oxidative damage to lung tissue. Therefore, Ceria-h exposure caused two waves of lung injury: BAL inflammation and cytotoxicity induced by particle overload in the early stage, and pulmonary lipid peroxidation and pro-inflammation in the latter stage. Since the intratracheal instillation of Ceria-h caused a more persistent lung injury compared to Ceria-p, the long-term pulmonary and systemic toxicity of Ceria-h needs further study.

Our results implied that the agglomeration, as well as the surface chemistry plays an important role in the pulmonary toxicity of nano-ceria. Due to agglomeration, Ceria-p did not exert more severe pulmonary toxicity when compared to nano-ceria sized 6.6 nm. Therefore, the characterization of the size distribution of the ceria-contained agglomerates in the exhaust is essential to assess the potential risks of using nano-ceria as a fuel additive. Meanwhile, more attention should be paid to the surface chemistry of nano-ceria after combustion. The ability to reversibly switch between Ce^3+^ and Ce^4+^ makes nano-ceria attractive as a fuel additive to reduce the release of greenhouse gases and particles. Meanwhile, nano-ceria are being proven promising for their SOD mimetic activity [21]. It was reported that nano-ceria with a high Ce^3+^/Ce^4+^ ratio on the surface could exhibit SOD mimetic activity, whereas nano-ceria with a lower Ce^3+^/Ce^4+^ ratio show no SOD mimetic activity [22]. In the present work, Ceria-p and Ceria-h both have no detectable fraction of Ce^3+^ (Figure 1B), thereby exhibiting negligible SOD mimetic activity (data not shown). The redox activity may also endow nano-ceria with the ability to catalyze the generation of ROS. Our previous work suggested that the oxidative stress caused by nano-ceria exposure at the environmentally relevant concentration would shorten worms’ lifespans [23]. Therefore, surface chemistry is also an important parameter to determine the pulmonary toxicity of the combusted nano-ceria. Unfortunately, so far, there was only very limited and controversial information on the properties of the combusted nano-ceria in the exhaust.

## 4. Subjects and Methods

### 4.1. Synthesis and Characterization of Nano-Ceria

The primary particle diameters of the two types of nano-ceria used in the present work were both 3–5 nm. One of the nano-ceria (Ceria-p) was synthesized by a precipitation method [24]. In brief, 1.736 g Ce(NO_3_)_3_ was added to NaOH solution (0.4 g NaOH dissolved in 128 mL water) followed by 48 h of magnetic stirring. The resulting white precipitate was collected and washed several times in ultrapure water.

Another type of nano-ceria (Ceria-h) was synthesized by a surfactant-assisted hydrothermal approach [25]. Briefly, 15 mL of 116.7 mmol/L Ce(NO_3_)_3_ solution was mixed with 15 mL toluene; then, 22.5 mL of 233.3 mmol/L sodium oleate aqueous was dropped into the above mixture solution with magnetic stirring. The upper layer toluene with cerium precursor was transferred to a 50-mL Teflon-lined stainless-steel autoclave with 15 mL deionized water and 0.35 mL *tert*-butylamine. The sealed autoclave was transferred to a 180 °C oven, held there for 12 h and then cooled to room temperature. Then, the brown supernatant solution with synthesized nano-ceria was precipitated with an adequate volume of ethanol. The precipitate was obtained by centrifugation for 10 min and re-dispersed in 15 mL ultrapure water containing 400 μL of 3-mercaptopropionic acid (3-MPA). After centrifuging and washing 3 times, a Ceria-p suspension was obtained.

The physicochemical properties of nano-ceria were characterized by transmission electron microscopy (TEM, JEM-2010, JEOL, Tokyo, Japan), dynamic light scattering (DLS, Zetasizer nano-ZS 90, Malvern Instrument, Worcestershire, UK), X-ray absorption near-edge structure (XANES, 1W1B beamline at Beijing Synchrotron Radiation Facility, Beijing, China), and X-ray diffraction (XRD, X’pert PRO MPD, PANalytical, Almelo, The Netherlands). The ROS generation catalyzed by nano-ceria was determined by the method described by the literature of Heckert [26], using 2,2’-azinobis-(3-ethylbenzthiazoline-6-sulfonic acid) (ABTS) as the free radical capture agent. After catching ROS, the ABTS can form a stable blue-green product, cation radicals ABTS^+#^, which was measured at 405 nm by using a UV-vis spectrophotometer (TU-1901, PGENENAL, Beijing, China). Reactions with nano-ceria were buffered by Tris (100 μM, pH 7.0). The solution containing 100 μM nano-CeO_2_, 88 mM H_2_O_2_ and 100 μM ABTS was added into a 1-cm path length quartz cuvette, and the absorbance at 405 nm was recorded once every 3 s for 10 min.

### 4.2. Animals

Male CD-1 (ICR) mice (6 weeks old) were purchased from Beijing Vital River Laboratories. A commercial pellet diet and deionized water were available *ad libitum*. After one week of acclimation, mice were randomly divided into 12 groups, with 8 mice per group and housed individually in stainless steel cages under standard laboratory conditions (temperature 23 ± 1 °C, humidity 50% ± 3% and a 12-h light/dark cycle). All the animal experiments were performed with the approval of the Ethics Committee of Animal Care and Experimentation of the National Institute for Environmental Studies, China.

### 4.3. Intratracheal Instillation of Nano-Ceria

The Ceria-p and Ceria-h were dispersed in ultrapure water at a concentration of 0.8 mg/mL, respectively, and sonicated for 15 min before use. By using a non-surgical intratracheal instillation method [8], mice were intratracheally instilled with 40 μg of either nano-ceria (50 μL of nano-ceria suspension) followed by 800 μL of air in 2 s. The control group was intratracheally instilled with ultrapure water instead of nano-ceria.

### 4.4. Cytological and Biochemical Analysis of Bronchoalveolar Lavage Fluid

The animals were sacrificed at 1, 3, 7 and 28 days post-instillation (PI). Bronchoalveolar lavage was performed 3 times with sodium phosphate buffer (PBS, pH 7.4, 1 mL for each lavage), and the cells in BALF were collected by centrifugation at 400× *g* and 4 °C for 10 min. The supernatant was used for biochemical analyses, while the recovered cells from the pellet were resuspended for the macrophage, neutrophil and lymphocyte cell counting with Wright’s staining. The activities of lactate dehydrogenase (LDH) and acid phosphatase (ACP) in the BALF were analyzed using colorimetric assay kits (Nanjing Jiancheng Bioengineering Institute, Nanjing, China). The concentration of total protein (TP) was determined by the bicinchoninic acid (BCA) method using a BCA protein assay kit (Thermo Fisher Scientific Incorporated, Rockford, IL, USA) with bovine serum albumin as a standard.

### 4.5. Biochemical Assay of Lung Homogenates

The lung tissues were separated and homogenized by a glass homogenizer in pre-cooled 0.01 M PBS (pH 7.4, *v*/*v* = 1/4). The homogenates were centrifuged at 10,000 rpm for 10 min at 4 °C, and the supernatants were aliquoted and stored at −80 °C for the biochemical analysis. The concentrations of malondialdehyde (MDA) and GSH, as well as the activities of SOD, GSH-Px and CAT in the lung homogenates were measured using the reagent kits purchased from Jiancheng Bioengineering Co., Ltd., Nanjing, China, according to the manufacturer’s protocols. Protein levels in the lung homogenates were determined by the bicinchoninic acid (BCA) protein assay kit (Thermo Fisher Scientific Incorporated, Rockford, IL, USA). The levels of IL-6 in the lung homogenates were determined using commercially available ELISA kits (Wuhan Boster Biological Technology Co., Ltd., Wuhan, China).

### 4.6. Histopathological Examination

Three pieces of lungs were selected randomly in each group at each time point. The lung tissues were immersed in 10% formaldehyde solution and subsequently embedded into paraffin wax, then sectioned into 5 μm-thick slices and mounted on a glass microscope slide. Then, the slices were stained with HE dye and observed using an optical microscope (Leica DM4000M, Wetzlar, Germany).

### 4.7. Statistical Analyses

All data were expressed as the mean ± standard deviation (SD). Statistical analyses were conducted with SPSS 16.0 (SPSS Inc., Chicago, IL, USA) statistical software for Windows. A one-way analysis of variance (ANOVA) and Tukey’s test were carried out to illustrate the significant difference between the treatment, and a difference of * *p* < 0.05 was considered statistically significant.

## 5. Conclusions

In the present work, we investigated the pulmonary toxicities of two kinds of nano-ceria with the same primary size distribution. Both Ceria-p and Ceria-h induced oxidative stress, inflammatory responses and cytotoxicities in mice, but possibly via different mechanisms. The mean size of Ceria-p agglomerates was much smaller compared to Ceria-h, thereby causing more potent, acute inflammation, due to its higher concentration number of agglomerates and higher deposition rate deep in the lung. Ceria-h exposure caused two waves of lung injury: BAL inflammation and cytotoxicity in the early stage and redox-activity-evoked lipid peroxidation, as well as pro-inflammation in the latter stage. Therefore, agglomerates size, as well as the surface chemistry of the combusted nano-ceria in the exhaust are essential characteristics to assess the potential risks of using nano-ceria as a fuel additive.
